# The Hall technique: knowledge, practice, and concerns of dentists in primary care settings in the State of Qatar—a questionnaire-based survey

**DOI:** 10.1007/s40368-024-00943-1

**Published:** 2024-10-04

**Authors:** H. A. Mohamed, E. M. Abdalla, N. A. HagOmer, N. Philip

**Affiliations:** 1 Primary Health Care Corporation, Doha, Qatar; 2 College of Dental Medicine, QU Health, Qatar University, Office 160C, Building H12, Doha, Qatar

**Keywords:** Hall technique, Preformed metal crowns, Pediatric dentists, General dental practitioners

## Abstract

**Purpose:**

To assess the knowledge, practice, and concerns about the Hall Technique among pediatric dentists (PDs) and general dental practitioners (GDPs) working in primary care settings in the State of Qatar.

**Methods:**

A prospective questionnaire-based survey was distributed to all GDPs and PDs working at different Primary Health Care Corporation centers in Qatar between 1 November and 10 December 2023. Descriptive data analysis was carried out.

**Results:**

The response rate was 49% among GPDs and 100% among PDs. Approximately 85% of PDs and 48% of GDPs were familiar with the HT although only 58% of the PDs and 4% of the GDPs applied HT preformed metal crowns (HTPMCs) in their current primary care practice. Majority of both PDs and GDPs did not consider HTPMCs as the treatment of choice for restoring asymptomatic carious primary molars and preferred applying the HT for cavitated carious lesions over non-cavitated lesions. Regarding HT practices, 57.7% of PDs and 48% of GDPs always planned on taking a pre-operative radiograph before the procedure, while only 15.4% of PDs and 8% of GDPs would always consider using an orthodontic separator prior to placing an HTPMC. The main concerns among the respondents about the HT included sealing in caries (PDs 31%; GDPs 50%), high occlusion (PDs 57.7%; GDPs 53.3%), and gingival damage (27% PDs; 47% GDPs.) Endorsement by professional bodies and further research evidence were seen as ways to promote wider adoption of the HT.

**Conclusion:**

The HT is recognised but not widely used among primary care dentists working in the State of Qatar. HTPMCs are often seen as secondary options for restoring carious primary molars with dentists still having a number of concerns regarding the HT.

## Introduction

The State of Qatar is among the richest countries in the world with the population having excellent access to medical and dental care. However, dental caries remains a major public health concern for children living in Qatar with national prevalence data suggesting that the disease affects up to 70–90% of 4–8-year-old children (Alkhtib et al. 2016; Al-Thani et al. 2018; Chrisopoulos et al. 2024). Childhood dental caries not only has a significant effect on the oral health of young children but also on their quality of life and that of their families too. Dental caries is among the commonest reasons for treatment under general anaesthesia for children in Qatar, posing a significant economic burden to individuals, the health sector, and society in general. There is a clear need for more conservative treatment options for carious lesions affecting primary teeth that are evidence-based, easier for the child (and parent) to cope with, and can be quickly implemented by general dental practitioners (GDPs) and pediatric dentists (PDs) working in primary care settings.

The ‘Hall Technique’ (HT), originally pioneered in 1997 by a GDP (Dr. Norna Hall) working in a high caries-risk area of Scotland, meets all the required criteria for conservative management of single or multi-surface asymptomatic carious primary molars (Welbury 2017). Traditionally, such teeth were managed by GDPs and PDs using amalgam/composite restorations or conventional preformed metal crowns (PMCs). However, these invasive and demanding conventional restorative techniques require local anaesthesia and technically challenging tooth preparation steps that are not always practical in young children. The HT on the other hand involves cementing an appropriately-sized PMC over carious primary molars without the use of rotary instruments and dental anaesthesia, usually after a five-to seven-day period of orthodontic separator placement to create space mesial and distal to the affected tooth (Innes et al. 2006, 2007). The HT can be used on children as young as three years and has been described as being the biggest revolution in paediatric dentistry (Deery and Doherty 2014). The HT is a truly biological technique based on the scientific rationale of sealing in the carious lesion rather than removing it, and works by depriving the cariogenic biofilm access to dietary carbohydrates thereby arresting lesion progression (Innes et al. 2017; Philip and Suneja 2023). This radical treatment option gave rise to a great deal of controversy and concerns in the past (Nainar 2012; Erdemci et al. 2014; Croll 2015), but as a seminal paper on the HT asserts ‘emotion, misinformation, and outdated ideas have been used in arguments against Hall crowns rather than logic, understanding or evidence’ (Innes et al. 2017). There is now robust evidence from randomised controlled trials (RCTs) demonstrating higher success for HT compared to non-restorative caries treatment, conventional amalgam, and resin-based restorations (Innes et al. 2011; Santamaria et al. 2014; Boyd et al. 2018; Pascareli-Carlos et al. 2023; Undre et al. 2023). RCTs have also shown similar success rates for the HT and conventionally-placed PMCs (Ludwig et al. 2014; Elamin et al. 2019; Midani et al. 2019; Ayedun et al. 2021). Several systematic reviews and meta-analyses have confirmed the superiority of HT compared to conventional restorative techniques (Innes et al. 2015a; Badar et al. 2019; Chua et al. 2023). Moreover, there is a high acceptability of HTPMCs among children, parents, and clinicians (Bell et al. 2010; Innes et al. 2011; Page et al. 2014; Santamaria et al. 2015; Almaghrabi et al. 2022). Given this overwhelming evidence, clinicians still reluctant to offer HT as a treatment option for asymptomatic carious primary molars, need to examine why they are treating a child more invasively when a less invasive successful option is available (Philip et al. 2018).

HTPMCs are widely used among PDs working in the UK with 96% reporting they used HTPMCs in their practice (Roberts et al. 2018). In the US, although a substantial portion of the PDs were familiar with the HT fewer than half used it (Gonzalez et al. 2021). Similarly, a global survey among PDs across 60 countries reported high awareness of HT, although only half the PDs provided HTPMCs in their regular practice (Hussein et al. 2020).

The HT may be particularly suitable in the primary care dental services of Qatar where a large majority of children are treated by GDPs and PDs. To the best of our knowledge, there has been no formal survey on the views, attitudes and usage of HTPMCs among GDPs and PDs working at various health centers of the Primary Health Care Corporation (PHCC) of Qatar. The aim of this study was to assess the knowledge, practice, and concerns about HTPMCs among GDPs and PDs of the PHCC in Qatar and their views on the perceived place of the HT for treating carious primary molars in children.

## Methods

This study was a cross-sectional questionnaire-based online survey that was distributed to GDPs and PDs working at different PHCC centers in Qatar. The questionnaire was adapted from previously published and validated surveys used in the UK and USA (Roberts et al. 2018; Gonzalez et al. 2021). Questions related to the application of HT under general anaesthesia or for medically compromised patients were excluded as they fell outside the scope of primary care settings. Prior to the application of the questionnaire, a pilot study was conducted to test its suitability in Qatar. After evaluating the responses of ten dentists in the pilot study, the questionnaire was considered appropriate without any modifications. Ethical approval for the study was obtained from the PHCC Research Committee of Clinical Research and Ethics (Buhooth-D-23-00074). A covering letter was sent with the survey, explaining why it was being done, with participation entirely voluntary and anonymous. The only inclusion criteria were that the participant is a GDP or PD working at the PHCC of Qatar. The survey was open from 1 November 2023 to 10 December 2023 with three reminders sent to potential participants over this period.

In the questionnaire, closed response modes were used, with either binary or multiple-choice or Likert-scale responses to each question. The questionnaire comprised of 22 multiple-choice questions divided into four sections: (i) demographics; (ii) knowledge about HTPMCs; (iii) practice and utilization of HTPMCs; and (iv) concerns and acceptability of the HT. Demographic information gathered included gender, graduation year, and professional designation (GDP or PD). Knowledge about HTPMCs was assessed through six questions covering familiarity with the HT, learning resources for HTPMCs, preferences for additional training on the HT, and opinions on the target audience for teaching the HT. The third section included questions about practitioners’ current usage of HTPMCs, how long they had been using the HT, whether the HTPMCs were a treatment option or the treatment of choice for different types of carious lesions in primary teeth, and the clinical conditions for using the HT (cavitated/non-cavitated, occlusal/proximal lesions). In this section, respondents were also asked whether they used pre-operative radiographs and orthodontic separators prior to applying HTPMCs, and also their assessment of the technique difficulty. The final section of the questionnaire addressed clinicians concerns about the HT and suggestions to encourage the use of HTPMCs. Questionnaire data was organised using a spreadsheet (Microsoft Excel 365, Redwood, USA). Data entered did not include the names of the respondent dentists. Descriptive data analysis was carried out using SPSS 24 (IBM, New York, USA).

## Results

### Study population demographics

The questionnaire survey was sent to 153 GDPs and 26 PDs working at the PHCC in Qatar. Completed responses were received from 75 GDPs (response rate 49%) and from all the PDs (response rate 100%). There was near equal gender distribution among both GDPs and PDs. Majority of the respondents among both GDPs and PDs were those who graduated before 2010 (Table 1).

**Table 1:** Survey participants demographics and knowledge about HTPMCs

Survey item	GDPs (*N* = 75) *N* (%)	PDs (*N* = 26) *N* (%)
*Gender*		
Male	37 (49.3%)	15 (57.7%)
Female	38 (50.7%)	11 (42.3%)
*Year of graduation*		
Before 2010	62 (82.7%)	18 (69.2%)
2010 or later	13 (17.3%)	8 (30.8%)
*Familiarity with HTPMCs*		
Very familiar	8 (10.7%)	13 (50%)
Familiar	28 (37.3%)	9 (34.6%)
Somewhat familiar	23 (30.7%)	3 (11.5%)
Unfamiliar	14 (18.7%)	0 (0%)
Very unfamiliar	2 (2.7%)	1 (3.8%)
*Learning resources about HTPMCs*		
Continuing dental education courses	36 (48%)	12 (46.2%)
Undergraduate dental school	21 (28%)	7 (26.9%)
Postgraduate residency	5 (6.7%)	18 (69.2%)
Scientific journals/online forums	19 (25.3%)	11 (42.3%)
Others (e.g. exam preparations)	1 (1.3%)	0 (0%)
Not familiar with HTPMCs	17 (22.7%)	0 (0%)
*To whom should HTPMCs be taught?*		
Undergraduate dental students	7 (9.3%)	3 (11.5%)
Postgraduate dental students	5 (6.7%)	3 (11.5%)
Both undergraduate and postgraduate students	57 (76%)	20 (76.9%)
Should not be taught	6 (8%)	0 (0%)
*Would you like to receive additional training on HTPMCs?*		
Yes	53 (70.7%)	11 (42.3%)
No	11 (14.7%)	10 (38.5%)
Not sure	11 (14.7%)	5 (19.2%)
*Which would be your preferred mode of additional training?*		
Webinars	31 (41.3%)	4 (15.4%)
Demonstrations at work	39 (52%)	10 (38.5%)
Video demonstrations	34 (45.3%)	11 (42.3%)
Hand-on-courses	51 (68%)	18 (69.2%)
Illustrated guidelines	15 (20%)	4 (15.4%)
Lectures	31 (41.3%)	12 (46.2%)

*HTPMC* Hall technique preformed metal crowns, *GDP* General Dental Practitioner; *PD* Paediatric Dentist

### Knowledge about HTPMCs

Approximately 85% of the PDs and 48% of the GDPs rated their knowledge about HTPMCs as either "very familiar" or "familiar". Post-graduate residency programs and continuing education courses were the main HT learning resources for PDs and GDPs respectively (Table 1). Over 7 in 10 GDPs expressed interest in receiving additional training on HTPMCs with the majority preferring hand-on-courses. Over 76% of PDs and GDPs agreed that the HT should be included in both the undergraduate and postgraduate curricula for dental students.

### Practice and utilization of HTPMCs

Only 4% of the GDPs were currently using the HT in their clinical practice while approximately 58% of the PDs applied HTPMCs in their current primary care practice (Table 2). Among PDs, 23.1% reported using HTPMCs for over 5 years, while another 23% of the PDs said they have been using the HT for two years or less. Majority of both PDs and GDPs considered HTPMCs as one of the treatment options and not the treatment of choice for restoring asymptomatic carious primary molars. Regarding HT practices, 57.7% of PDs and 48% of GDPs always plan on taking a pre-operative radiograph before the procedure, while only 15.4% of PDs and 8% of GDPs would always consider using an orthodontic separator prior to placing an HTPMC. Approximately 65% of PDs and 67% of GDPs rarely or never used the HT when the clinical diagnosis was a non-cavitated occlusal carious lesion, while more than 73% of the PDs and 57% of the GDPs would often or sometimes consider HTPMCs to restore cavitated occlusal carious lesions. Over 57% of PDs and 46% of GDPs would always or often consider restoring cavitated inter-proximal lesions with HTPMCs. Majority of the PDs (76.9%) found the HT manageable or easy to handle compared to 41% of GDPs. A little over 73% of PDs reported moderate to no apparent discomfort for children during the HTPMC procedure, compared to 49.4% of GDPs.

**Table 2:** Practice and utilization of HTPMCs among GDPs and PDs

Survey Item	GDPs (*N* = 75) *N* (%)	PDs (*N* = 26) *N* (%)
*Do you currently use HTPMCs in your primary care practice?*		
Yes	3 (4%)	15 (57.7%)
No	72 (96%)	11 (42.3%)
*When would you use or consider to use HTPMCs?*		
Treatment option for symptomless carious primary molar	13 (17.3%)	21 (80.8%)
Treatment of choice for symptomless carious primary molar	3 (4%)	0 (0%)
Only when unable to use a conventional restoration in a carious primary molar	11 (14.7%)	1 (3.8%)
I do not use HTPMCs	48 (64%)	4 (15.4%)
*Would you take a preoperative radiograph prior to placement of HTPMCs?*		
Always	36 (48%)	15 (57.7%)
Often	8 (10.7%)	4 (15.4%)
Sometimes	16 (21.3%)	6 (23.1%)
Rarely	3 (4%)	0 (0%)
Never	12 (16%)	1 (3.8%)
*Would you use orthodontic separators prior to placement of HTPMCs?*		
Always	6 (8%)	4 (15.4%)
Often	7 (9.3%)	7 (26.9%)
Sometimes	25 (33.3%)	13 (50%)
Rarely	10 (13.3%)	0 (0%)
Never	27 (36%)	2 (7.7%)
*Would you plan to use HTPMCs for a non-cavitated occlusal carious lesion?*		
Always	2 (2.7%)	1 (3.8%)
Often	9 (12%)	1 (3.8%)
Sometimes	14 (18.7%)	7 (26.9%)
Rarely	6 (8%)	7 (26.9%)
Never	44 (58.7%)	10 (38.5%)
*Would you plan to use HTPMCs for a cavitated occlusal carious lesion?*		
Always	10 (13.3%)	2 (7.7%)
Often	17 (22.7%)	9 (34.6%)
Sometimes	26 (34.7%)	10 (38.5%)
Rarely	5 (6.7%)	3 (11.5%)
Never	17 (22.7%)	2 (7.7%)
*Would you plan to use HTPMCs for an apparently non-cavitated interproximal carious lesion?*		
Always	8 (10.7%)	4 (15.4%)
Often	4 (5.3%)	3 (11.5%)
Sometimes	25 (33.3%)	7 (26.9%)
Rarely	8 (10.7%)	4 (15.4%)
Never	30 (40%)	3 (11.5%)
*Would you plan to use HTPMCs for a cavitated interproximal carious lesion?*		
Always	15 (20%)	6 (23.1%)
Often	20 (26.7%)	9 (34.6%)
Sometimes	21 (28%)	9 (34.6%)
Rarely	3 (4%)	1 (3.8%)
Never	16 (21.3%)	1 (3.8%)
*Technique difficulty of HTPMCs*		
Very easy to handle	1 (1.3%)	1 (3.8%)
Easy to handle	8 (10.7%)	8 (30.8%)
Manageable	22 (29.3%)	11 (42.3%)
Difficult to handle	6 (8%)	2 (7.7%)
I never used HTPMCs	38 (50.7%)	4 (15.4%)

*HTPMC* Hall technique preformed metal crowns, *GDP* General Dental Practitioner, *PD* Paediatric Dentist

### Concerns and acceptability of the HT

Regarding concerns associated with HT, over 50% of the GDPs agreed or strongly agreed with concerns about sealing in caries, compared to 31% of PDs who expressed similar concerns (Table 3). Regarding high occlusion associated with HTPMCs, both PDs and GDPs shared this concern (57.7% of PDs and 53.3% of GDPs). Lower numbers, 27% of PDs and 47% of GDPs, agreed or strongly agreed with concerns about gingival damage when using the HT. Over 73% of the PDs and 65% of the GDPs agreed or strongly agreed that endorsement of the HT by either the American Academy of Pediatric Dentistry (AAPD) or the European Academy of Paediatric Dentistry (EAPD) would encourage the use of HTPMCs. Majority of both PDs and GDPs agreed or strongly agreed that additional research evidence would encourage wider use of HTPMCs.

**Table 3:** Concerns and acceptability of the Hall technique (HT)

Survey item	GDPs (*N* = 75) *N* (%)	PDs (*N* = 26) *N* (%)
*Concerns about sealing in decay during the HT*		
Strongly disagree	7 (9.3%)	8 (30.8%)
Disagree	14 (18.7%)	4 (15.4%)
Neither agree nor disagree	16 (21.3%)	6 (23.1%)
Agree	34 (45.3%)	7 (26.9%)
Strongly agree	4 (5.3%)	1 (3.8%)
*Concerns about high occlusion associated with the HT*		
Strongly disagree	1 (1.3%)	2 (7.7%)
Disagree	11 (14.7%)	2 (7.7%)
Neither agree nor disagree	15 (20%)	7 (26.9%)
Agree	40 (53.3%)	15 (57.7%)
Strongly agree	8 (10.7%)	0 (0%)
*Concerns about gingival tissue damage associated with the HT*		
Strongly disagree	3 (4%)	4 (15.4%)
Disagree	15 (20%)	6 (23.1%)
Neither agree nor disagree	22 (29.3%)	9 (34.6%)
Agree	28 (37.3%)	6 (23.1%)
Strongly agree	7 (9.3%)	1 (3.8%)
*Concerns about eruption interference associated with the HT*		
Strongly disagree	2 (2.7%)	7 (26.9%)
Disagree	28 (37.3%)	9 (34.6%)
Neither agree nor disagree	18 (24%)	8 (30.8%)
Agree	21 (28%)	2 (7.7%)
Strongly agree	6 (8%)	0 (0%)
*Concerns about lack of confidence in current evidence on the HT*		
Strongly disagree	5 (6.7%)	5 (19.2%)
Disagree	19 (25.3%)	10 (38.5%)
Neither agree nor disagree	26 (34.7%)	8 (30.8%)
Agree	22 (29.3%)	3 (11.5%)
Strongly agree	3 (4%)	0 (0%)
*Would AAPD/EAPD endorsement of the HT encourage its use?*		
Strongly disagree	4 (5.3%)	0 (0%)
Disagree	1 (1.3%)	0 (0%)
Neither agree nor disagree	21 (28%)	7 (26.9%)
Agree	35 (46.7%)	13 (50%)
Strongly agree	14 (18.7%)	6 (23.1%)
*Would additional research evidence encourage use of the HT?*		
Strongly disagree	4 (5.3%)	0 (0%)
Disagree	1 (1.3%)	1 (3.8%)
Neither agree nor disagree	19 (25.3%)	4 (15.4%)
Agree	41 (54.7%)	17 (65.4%)
Strongly agree	10 (13.3%)	4 (15.4%)

*GDP* General Dental Practitioner, *PD* Paediatric Dentist, *AAPD* American Academy of Pediatric Dentistry, *EAPD* European Academy of Paediatric Dentistry

## Discussion

The HT has outperformed other restorative techniques in randomised clinical trials and systematic reviews and therefore greater consideration must be given towards using the HT for managing carious primary molars (Chua et al. 2023). In Qatar, child dental care is mostly delivered by GDPs and PDs working in primary care settings, making it critical to assess their knowledge, practices, and concerns about HT. The results of this questionnaire-based survey indicate that nearly all the PDs and over 70% of the GDPs in Qatar were familiar with the HT which is in line with previous surveys conducted in the UK (Roberts et al. 2018), US (Gonzalez et al. 2021), Scotland (Dean et al. 2011), and globally (Hussein et al. 2020), but in contrast to German dentists (Santamaría et al. 2018), majority of whom were not familiar with the HT. However, the use of HT in Qatar primary care settings did not match the knowledge, with less than 6 in 10 of the PDs and only 3 out of 76 GDPs currently using it in their practice. For PDs, these numbers on current usage of the HT are similar to those reported globally among PDs (Hussein et al. 2020), but much lower than in the UK (Roberts et al. 2018) where 96% of the PDs use the HT. The reasons why familiarity with the HT did not match current usage could possibly be due lack of firm recommendations about its use from bodies like the AAPD and EAPD, an incomplete understanding of contemporary cariology concepts, and practitioners being more familiar with traditional restorative techniques.

Almost 70% of the PDs in this study reported that they learned about HT only during their post-graduate residency programs, probably because 69% of the PDs in this study obtained their basic dental qualification before 2010. Continuing dental education courses about HT was the most common source of knowledge among the GDPs. Majority of both GDPs and PDs in Qatar agreed that the HT should be taught both to undergraduate students and postgraduate residents similar to other studies (Roberts et al. 2018; Hussein et al. 2020), although a substantial proportion of US-based PDs suggested that the HT should be taught only to postgraduate residents (Gonzalez et al. 2021). The robust evidence base for the HT suggests that this less invasive caries management approach should be taught to all dental students as part of the standard treatment protocol for carious primary molars, rather than restricting the teaching of the HT to only specialist training. Significant majorities of both GDPs and PDs surveyed in this study showed interest in additional training on the HT, preferably via hands-on-courses, indicating their interest in applying this technique in their regular primary care practice.

The results of this study suggest that HTPMCs are not used by vast majorities of both GDPs and PDs as the treatment of choice for symptomless carious primary molars. Instead GDPs and PDs in Qatar viewed HTPMCs as only one of the treatment options to restore carious primary molars. These findings are similar to those reported elsewhere (Roberts et al. 2018; Hussein et al. 2020). It is not clear why HTPMCs are not the treatment of choice despite clinical trials reporting success rates over 90%, their ease of fitting, and high acceptability among child patients (Innes et al. 2011; Ludwig et al. 2014; Santamaria et al. 2014; Boyd et al. 2018; Elamin et al. 2019; Midani et al. 2019; Ayedun et al. 2021; Pascareli-Carlos et al. 2023; Undre et al. 2023). It is possible that HTPMCs will over time become the treatment of choice to restore indicated carious primary molars once more evidence accumulates and it is used more often by dentists in their regular practice. This study also indicates that both GDPs and PDs plan to use HTPMCs more often in cavitated lesions than non-cavitated lesions. This is in keeping with current guidelines where non-cavitated occlusal/proximal lesions are recommended to be treated by measures like fissure sealants, fluoride varnish application, or resin infiltration (Philip and Suneja 2023). Moreover, the HT manual (Innes et al. 2015b) also advises the use of HTPMCs for cavitated two-surface lesions or extensive one-surface lesions. The HT manual also recommends that obtaining a preoperative radiograph prior to placing HTPMCs is best practice so as to confirm that a clear layer of dentine is present between the lesion floor and the pulp. Most dentists surveyed in this study appear to indicate that they would take a preoperative radiograph prior to fitting a HTPMC which is in keeping with the HT manual recommendation. If the child is uncooperative or unable to tolerate bitewings, clinicians can possibly place an HTPMC provided they rule out any carious pulp involvement and the tooth is clinically symptomless (Roberts et al. 2018). Majority of the PDs in this study also indicated that they would consider the use of orthodontic separators before the HT to create sufficient proximal space. This agrees with other studies that surveyed PDs on the use of orthodontic separators before applying the HT (Roberts et al. 2018; Hussein et al. 2020) and is the recommended norm (Innes et al. 2009). Although the ‘modified HT’, where high-speed preparations are used to open up proximal spaces is gaining popularity (Clark et al. 2017) and has also been reported to be as successful as the traditional HT (Midani et al. 2019), it contradicts the HT manual and the rationale of this less invasive treatment approach.

GDPs and PDs surveyed in this study expressed varying degrees of concern with regard to the different aspects of the HT. Sealing in caries under the Hall crown potentially leading to pulp necrosis was a concern expressed more by GDPs than PDs, although 8 out of the 26 PDs surveyed also expressed similar concerns. Similar doubts have been raised by US-based PDs regarding pulp necrosis that may occur under Hall crowns (Gonzalez et al. 2021). The biological rationale of the HT is to seal the carious lesion under the PMC to isolate the lesion from the cariogenic oral environment, deprive the cariogenic biofilm of nutrients, and thereby inactivate it. Multiple randomised controlled trials and systematic reviews have not found any detrimental effects to the pulp by sealing in and isolating the carious lesion as happens when placing HTPMCs (Mertz-Fairhurst et al. 1998; Innes et al. 2011; Ricketts et al. 2013; Hesse et al. 2014). Another commonly cited concern about the HT, shared equally by both GDPs and PDs, was the increase in the occlusal vertical dimension after placing the Hall crown. However, several clinical studies have shown that the initial high occlusion experienced after placing the Hall crown returns to pre-treatment levels within four weeks (van der Zee and van Amerongen 2010; Santamaria et al. 2014; Joseph et al. 2020). There were also concerns among study respondents about the gingival health around the tooth when using the HT. However, a randomised controlled trial comparing HTPMCs and conventional PMCs found no difference in their plaque or gingival index with both measures improving over the course of the study (Elamin et al. 2019). Furthermore, there were some concerns among GDPs and PDs about possible eruption interferences associated with HTPMCs similar to those reported previously (Clark et al. 2017; Gonzalez et al. 2021). Regular reviews and radiographic evaluations post placement of HTPMCs can identify and address eruption interferences promptly. Finally, a few dentists also expressed a lack of confidence in the current evidence on the HT. However, this may be due to busy clinicians in primary care settings not being fully updated with regard to the now overwhelming evidence available on the highly successful treatment outcomes of HTPMCs. Participants of this study indicated that endorsements of the HT by the AAPD or EAPD and additional research evidence could encourage the future use of HTPMCs. Currently, HT is mentioned in the AAPD’s reference manual (AAPD 2020) but without any firm recommendations about its indications and best practices.

The main limitation of this questionnaire-based survey was the relatively low response rate received from GDPs working in the Qatar PHCC clinics. It is also likely that GDPs who responded to the survey may be more interested in the HT than those who did not respond, further affecting the overall outcome of this study. Secondly, the survey was limited only to dentists working in the PHCC clinics, and thus its results may not necessarily reflect the opinions of the larger majority of GDPs and PDs working in the private health sector and secondary care institutions of the country. Finally, the responses received to the survey questions are prone to recall bias as they were reliant on the respondents’ memory.

Despite the accumulating evidence, significant barriers to translating knowledge and practice of HT into primary care settings are expected to remain. These barriers include clinician resistance to adopting new practices, inconsistencies in training and experience, and inadequate understanding of contemporary cariology concepts. Overcoming these obstacles will necessitate comprehensive strategies, including continuing professional education and more robust evidence-based clinical trials. Such efforts are essential to facilitate the integration of HT into routine primary care dental practice and ensure its effective utilization in managing carious lesions of primary molars.

## Conclusion

HTPMCs are well recognized by the majority of respondents in this study. However, only 4% of the GDPs and approximately 58% of the PDs in the Qatar PHCC clinics reported using the HT in regular practice. The main impediments to adopting HTPMCs include insufficient training, concerns about the high occlusion of Hall crowns, the perception that it constitutes poor quality dentistry, and the need for more supporting high-quality clinical evidence. However, the majority of respondents believe that HT should be incorporated into the curricula for both undergraduate and postgraduate dental students and await official endorsement of the HT by the AAPD and the EAPD.

## Data Availability

The data that support the findings of this study are available from the corresponding author upon reasonable request.

## Code availability

Non-applicable.
